# Excretion of complement proteins and its activation marker C5b-9 in IgA nephropathy in relation to renal function

**DOI:** 10.1186/1471-2369-12-64

**Published:** 2011-11-23

**Authors:** Kisara Onda, Isao Ohsawa, Hiroyuki Ohi, Mariko Tamano, Satoshi Mano, Michiro Wakabayashi, Akie Toki, Satoshi Horikoshi, Teizo Fujita, Yasuhiko Tomino

**Affiliations:** 1 Division of Nephrology, Department of Internal Medicine, Juntendo University Faculty of Medicine, Tokyo, Japan; 2 Department of Immunology, Fukushima Medical University School of Medicine, Fukushima, Japan

## Abstract

**Background:**

Glomerular damage in IgA nephropathy (IgAN) is mediated by complement activation via the alternative and lectin pathways. Therefore, we focused on molecules stabilizing and regulating the alternative pathway C3 convertase in urine which might be associated with IgAN pathogenesis.

**Methods:**

Membrane attack complex (MAC), properdin (P), factor H (fH) and Complement receptor type 1 (CR1) were quantified in urine samples from 71 patients with IgAN and 72 healthy controls. Glomerular deposition of C5, fH and P was assessed using an immunofluorescence technique and correlated with histological severity of IgAN and clinical parameters. Fibrotic changes and glomerular sclerosis were evaluated in renal biopsy specimens.

**Results:**

Immunofluorescence studies revealed glomerular depositions of C5, fH and P in patients with IgAN. Urinary MAC, fH and P levels in IgAN patients were significantly higher than those in healthy controls (p < 0.001), but CR1 was significantly lower than that in healthy controls (p < 0.001). Urinary MAC and fH levels were positively correlated with serum creatinine (sCr), urinary N-acetyl-β-D-glucosaminidase (u-NAG), urinary β2 microglobulin (u-Bm), urinary protein (p < 0.001), interstitial fibrosis (MAC: p < 0.01, fH: p < 0.05) and the percentage of global glomerular sclerosis (p < 0.01). Urinary P was positively correlated with u-NAG, u-Bm, and urinary protein (p < 0.01).

**Conclusions:**

Complement activation occurs in the urinary space in IgAN and the measurement of levels of MAC and fH in the urine could be a useful indicator of renal injury in patients with IgAN.

## Background

IgA nephropathy (IgAN) is the most common form of glomerular disease worldwide. Predominant deposition of IgA1 and C3 in mesangial areas is accepted as a hallmark diagnostic feature of IgAN. Immunohistological findings on complement components showed deposits of C3 and properdin (P) in the glomerular mesangial areas and the absence of C1q in patients with IgAN [1-3]. Thus, it has been thought that the activation of the alternative pathway plays a crucial role in the pathogenesis of IgAN. However, recent studies revealed that 25% of patients with IgAN had mesangial deposits of mannose-binding lectin (MBL), L-ficolin, MBL-associated serine protease and C4, suggesting that the lectin pathway activation may also be important in some IgAN patients [4-7]. In any event, activation of C3 and C3 convertase production are the key causes of histological damage induced following membrane attack complex (MAC; C5b-9) formation. MAC is produced via the activated common terminal pathway of all three complement pathways.

There are several proteins which stabilize or regulate C3 convertase activation via the alternative or lectin pathways. C3bBb is an unstable form of C3 convertase with a half-life of 90 seconds. C3bBb associates with and is stabilized by P, to form the C3bBbP, with a half-life extended 5-10-fold [8]. Factor H (fH) plays a crucial role in inhibition of the alternative pathway by the following mechanism: 1) fH is a cofactor for factor I (fI) in cleaving C3b to inactivate C3bi [9,10] and 2) fH accelerates the decay of C3b, Bb, and C3bBbP [11]. Complement receptor type 1 (CR1; CD35) is a natural membrane-bound regulator and has specificity for C3b and C4b with the ability to displace the catalytic subunits from C3 or C5 convertase and to function as a co-factor for the degradation of C3b and C4b mediated by factor I [12,13].

Because our previous work established that the serum levels of B, P, fH and fI in patients with IgAN were significantly higher than those in healthy controls [14], we hypothesized that targeting the alternative pathway C3 convertase activation could be therapeutically beneficial in IgAN. In other types of glomerular disease, such as membranous nephropathy and lupus nephritis, patients' urine contains complement regulatory proteins and MAC, amounts of which fluctuate with disease activity [15-17]. Here, we investigated these issues using urine samples from patients with IgA nephropathy, which, unlike serum, can be obtained noninvasively.

## Methods

### Patients and controls

Seventy-one patients with IgAN (38 males and 33 females), who had been referred to Juntendo University Hospital between March 2003 and May 2005, were enrolled. Age of these patients at the time of urine collection ranged from 16 to 67 years old (37.8 ± 12.8, mean ± SD). Normal controls were 72 healthy volunteers (58 males and 14 females). This study was approved by the institutional human study Ethics Committee and informed consent was obtained before participation. Histological diagnosis was classified by standard examination of renal biopsy specimens by light microscopic findings with the results of immunoglobulin and complement deposition by immunofluorescence technique. According to the Japanese Clinical Guidelines for Patients with IgAN [18], patients were divided into four groups as follows: good prognosis, relatively good prognosis, relatively poor prognosis and poor prognosis (Table 1).

**Table 1:** Histological severity of IgAN (Japanese Clinical Guidelines )

	Mesangial cell proliferation and increased matrix	**Glomerulosclerosis**, crescent formation or adhesion to Bowman's capsule	**Interstitium**, renal tubuli or blood vessels
Good prognosis	Slight	Absent	Prominent changes are not seen

Relatively good prognosis	Slight	< 10% of all biopsied glomeruli	Prominent changes are not seen

Relatively poor prognosis	Moderate, diffuse	10-30% of all biopsied glomeruli	Cellular infiltration is slight in the interstitium except around some sclerosed glomeruli. Tubular atrophy is slight, and mild vascular sclerosis.

Poor prognosis	Severe, diffuse	> 30% of all biopsied glomeruli	Interstitial cellular infiltration and tubular atrophy, as well as fibrosis are seen. Hyperplasia or degeneration may be seen in some intrarenal arteriolar walls.

### Laboratory data

Serum total protein (TP), urinary protein (urinary protein (mg/dl)/urinary creatinine (mg/dl)), urinary N-acetyl-β-D-glucosaminidase (u-NAG), urinary β_2_-microglobulin (u-Bm) and serum levels of urea nitrogen (SUN), and creatinine (s-Cr) were measured as part of the routine clinical analyses at the time of urine collection. Laboratory data were undertaken at the central laboratory in the Juntendo University Hospital.

### Glomerular deposition of Immunoglobulins, C1q, C3, C5, fH and P

Renal biopsy specimens were frozen and examined by direct immunofluorescence staining, performed using fluorescein-5-isothiocyanate-labeled rabbit anti-human IgG, IgA, IgM, C1q and C3 antisera (Dako, Denmark), goat anti-human C5 and P antisera (Nordic Immunological Laboratories, Tilburg, Netherlands), and rabbit anti-human fH antiserum labeled by Linkit™ Fluoro-Link (ISL, Paignton, UK). IgG, IgA, IgM, C1q and C3 were diluted to 1:50 in 0.01 mol/l PBS, ph7.4, and C5, fH and P were diluted to 1:10 in the same buffer.

### Measurement of complement regulatory proteins and MAC in urine

Urine samples were obtained and stored at -80°C until use. Rabbit antisera to human fH, P, CR1, and purified P were kindly provided by Professor Teizo Fujita (Department of Biochemistry, Fukushima Medical University, Japan). Biotinylated anti-human properdin antibody was purchased from AntibodyShop (Gentofte, Denmark).

Urinary concentrations of fH and MAC were measured by commercially available sandwich enzyme-linked immunosorbent assay (ELISA) kits (BTA TRAK Kit, Alidex, Inc., Redmond, WA, USA, and SC5b-9 EIA, Quidel, San Diego, CA, USA). ELISA for P and CR1 were developed in our institute. Urinary P was quantified as described in our previous report [14]. CR1 in urine was determined using 4 μg/ml rabbit anti-human CR1 antibody and 0.1 μg/ml biotinylated mouse anti-human CR1 antibody (Ancell Corporation, Bayport, MN, USA) [19].

### Western blot analysis for urinary fH

To obtain a detectable amount of urinary fH, the urine samples were concentrated fourfold by Ultrafree-MC Centrifugal Filter Units (Millipore, Bedford, MA, USA). Urine samples were electrophoresed on 5% SDS-PAGE gradient gels under non-reducing conditions and the resultant bands were transferred to Immobilon™ (Millipore, Bedford, MA, USA). The immunoblots were incubated with biotinylated mouse anti-human fH antibody (AntibodyShop, Gentofte, Denmark), and incubated with streptavidin-peroxidase (Streptavidin-HRP, Southern Biotechnology Associates, Inc. Birmingham, AL, USA), and then developed using the ECL-plus system (Amersham Biosciences, Little Chalfont, UK).

### Evaluation of fibrotic changes and glomerular sclerosis

Fibrotic changes were evaluated on Azan and Masson-Trichrome-stained slides from 60 cases. Interstitial fibrosis was assessed by measuring the percentage of fibrotic (collagen-positive) area against whole area of specimen, using the KS400 Carl Zeiss image analysis system (KS400, Carl Zeiss Imaging Solutions GmbH, Hallbergmoos, Germany).

The percentage of global glomerular sclerosis as a fraction of all glomeruli was determined in 39 renal biopsy specimens by light microscopy.

### Statistical analysis

Data are shown as mean ± SD. Comparisons among the groups were performed by the Mann-Whitney U test, and comparisons of the four classifications were performed by the Bonferroni's Multiple Comparison test. Correlations among the groups were evaluated by linear regression. P values < 0.05 were considered significant in all analyses.

## Results

### Patients' background

All patients with IgAN were classified according to the Japanese Clinical Guidelines [18] and their clinical characteristics are shown in Table 2. U-NAG levels in the poor prognosis group were significantly higher than those in the other groups (p < 0.05). Significant differences in levels of TP, s-Cr and urinary protein were observed among the four groups. Regarding differences associated with disease severity, levels of urinary MAC were increased; especially in the poor prognosis group it was tending significantly higher than in the good prognosis group.

**Table 2:** Clinical characteristics of the patients

Classification (The number of biopsies and range of glomeruli)	TP (g/dl)	s-Cr (mg/dl)	u-Protein (g/g•Cr)	u-NAG (10^-3^U/mg•Cr)	u-Bm (ng/mg•Cr)	u-MAC (ng/mg•Cr)
Good prognosis (n = 6, 7.5 ± 4.5)	7.3 ± 0.6	0.64 ± 0.18	0.41 ± 0.19	3.6 ± 2.3*	57.7 ± 17.3	0.3 ± 0.8

Relatively good prognosis (n = 17, 15.3 ± 9.6)	7.4 ± 0.3**	0.80 ± 0.19	0.39 ± 0.36*	3.6 ± 2.0*	110.2 ± 113.1	6.5 ± 11.0

Relatively poor prognosis (n = 30, 13.0 ± 6.9)	7.0 ± 0.5	0.72 ± 0.23*	0.75 ± 0.67	4.5 ± 2.2*	222.9 ± 570.5	12.1 ± 24.9

Poor prognosis (n = 18, 14.9 ± 9.6)	6.8 ± 0.4	0.98 ± 0.48	1.10 ± 0.92	9.3 ± 6.7	254.4 ± 528.3	25.4 ± 42.7

Total (n = 71)	7.1 ± 0.5	0.80 ± 0.32	0.72 ± 0.70	5.4 ± 4.4	189.9 ± 458.4	14.0 ± 28.4

(mean ± SD)

*: p < 0.05 vs. poor prognosis,

**:p < 0.05 vs. relatively poor prognosis and poor prognosis

Abbreviations: TP, total protein; s-Cr, serum creatinine; u-Protein, urinary protein; u-NAG, urinary N-acetyl-β-D-glucosaminidase; u-Bm, u-β2 microglobulin; u-MAC, urinary membrane attack complex

### Glomerular deposition of complement components and regulatory proteins

**Figure 1:**
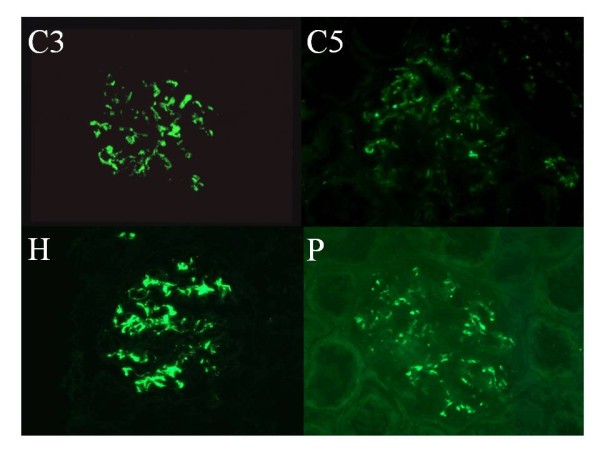
**Glomerular deposition of C3, C5, fH and P were assessed by immunofluorescence technique**. Glomerular deposition of C3, C5, fH and P shows the same mesangial pattern.

Immunofluorescence technique revealed deposits of C3, C5, fH and P in glomeruli of IgAN patients (Figure 1). The coarse granular deposits of all these factors appeared to have a similar mesangial distribution pattern. From these results, we inferred that the alternative pathway C3 convertase was activated and regulated in the IgAN glomeruli.

### Urinary MAC and complement regulatory proteins

**Figure 2:**
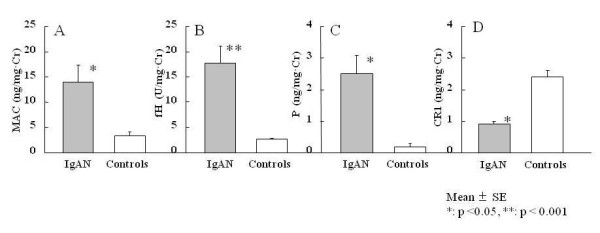
**Urinary MAC and complement regulatory protein levels differ between patients with IgA nephropathy and healthy controls**. Levels of urinary MAC, fH, and P are significantly higher in IgAN patients than those in healthy controls, but CR1 is significantly lower.

**Figure 3:**
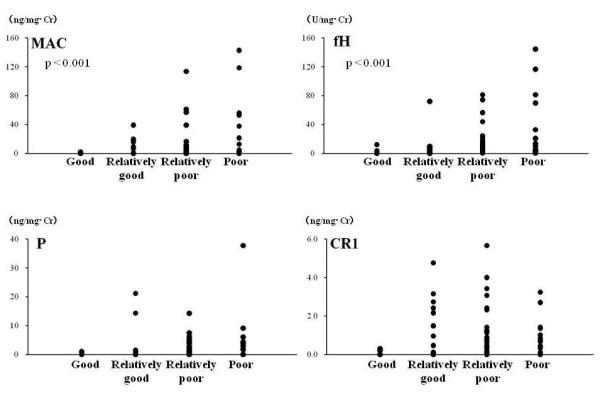
**Levels of urinary MAC and complement regulatory proteins in IgA nephropathy patients grouped according to disease severity**. Urinary MAC, fH and P tends to increase with worsening of prognosis, in particular, MAC and fH significantly fluctuate with disease prognosis (p < 0.001).

Urinary complement components in IgAN patients and healthy controls are shown in Figure 2. In IgAN patients, urinary MAC, fH, and P levels were significantly higher than those in healthy controls (Figure 2A, B, C), whereas urinary CR1 was significantly lower (Figure 2D). Figure 3 shows the relationship between urinary MAC, fH, P, CR1 and disease severity. In particular, levels of urinary MAC and fH significantly increased with increased disease severity (p < 0.001).

Correlations between urinary complement and clinical markers for renal disease were sought (Table 3). Urinary MAC was significantly correlated with s-Cr (p < 0.01), u-NAG (p < 0.001), u-Bm (p < 0.001) and urinary protein (p < 0.001). There was also a significant correlation between fH and all parameters (p < 0.001). Levels of urinary MAC and fH dovetail with clinical disease activity. Levels of urinary CR1 were significantly correlated with u-Bm (p < 0.01), but there was no significant correlation between CR1 and any other parameters.

**Table 3:** Correlations between levels of urinary MAC and complement regulatory proteins in IgAN patients

	MAC		fH		P		CR1	
	**r**	**p**	**r**	**p**	**r**	**p**	**r**	**p**

s-Cr	0.297	p < 0.01	0.531	p < 0.001	0.192	p = 0.102	-0.174	p = 0.137

u-NAG	0.589	p < 0.001	0.633	p < 0.001	0.419	p < 0.001	0.491	p = 0.481

u-Bm	0.414	p < 0.001	0.384	p < 0.001	0.367	p < 0.01	0.323	p < 0.01

u-Protein	0.458	p < 0.001	0.645	p < 0.001	0.502	p < 0.001	-0.181	p = 0.122

Abbreviations: MAC, membrane attack complex; fH, factor H; P, properdin; CR1, complement receptor 1; s-Cr, serum creatinine; u-NAG, urinary N-acetyl-β-D-glucosaminidase; u-Bm, u-β2 microglobulin; u-Protein, urinary protein

### Molecular weight of urinary fH

**Figure 4:**
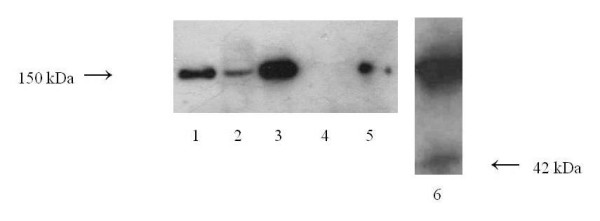
**Detection of fH in urine and serum samples by Western blotting**. Representative results show molecular weight assessments for fH. Lane 1: purified fH, lane 2: urine a from patient, 3: serum from the same patient, 4: urine from a healthy control, 5: serum from a healthy control, 6: urine from the patient with the highest level of urinary fH.

The molecular weight of urinary fH was evaluated by Western blotting in 7 patients with levels **>**50 U/mg·Cr (Figure 4). Serum fH had an estimated molecular weight of 150 kDa in all patients, as well as in the healthy controls. There was no fH in the urine of healthy controls. Urinary fH in IgAN patients was also 150 kDa, except for one patient whose urine had the highest level of fH and contained a 42 kDa protein (factor H like protein 1: FHL-1).

### Urinary complement and histological changes

Correlations between urinary complement levels and interstitial fibrosis, and between urinary complement levels and percentage of global glomerular sclerosis, were evaluated in IgAN patients (Table 4). Urinary MAC was significantly correlated with interstitial fibrosis (p < 0.01) and the percentage of global glomerular sclerosis as a fraction of all glomeruli (p < 0.01). Urinary fH levels were significantly correlated with interstitial fibrosis (p < 0.05) and the percentage of global glomerular sclerosis (p < 0.01).

**Table 4:** Correlations between urinary complement levels and fibrosis in IgAN patients

	Interstitial fibrosis			Percentage of global glomerular sclerosis as a fraction of all glomeruli	
	**r**	**p**		**r**	**p**

MAC	0.476	p < 0.01		0.361	p < 0.01
fH	0.383	p < 0.05		0.411	p < 0.01
P	0.310	p = 0.054		0.215	p = 0.099
CR1	-0.179	p = 0.279		-0.158	p = 0.229
U-protein	0.421	p < 0.01		0.339	p < 0.01

Abbreviations: MAC, membrane attack complex; fH, factor H; P, properdin; CR1, complement receptor 1; U-protein, Urinary protein

Significant correlations were also found between interstitial fibrosis and urinary protein (r = 0.421, p < 0.01), and between percentage of global glomerular sclerosis and urinary protein (r = 0.339, p < 0.01).

## Discussion

IgAN is the most common chronic glomerulonephritis with one third of patients developing progressive end-stage renal failure [20,21]. Although complement activation leads to tissue damage in IgAN, the role of complement regulatory proteins in the pathogenesis of IgAN has not been clearly defined. Our previous report and others documented increased serum levels of fH and P in IgAN patients and that serum levels of complement regulatory proteins reflected IgAN disease activity [14,22]. Based on these findings, we planned to evaluate the significance of urinary complement components in the pathogenesis of IgAN.

In other renal diseases, strong associations of urinary fH and MAC levels with disease progression have been demonstrated (15-17). Recently, Zang et al. proposed that urinary fH in patients with IgAN may be a useful biomarker to evaluate kidney injury [23]. In that report, the analysis was limited to urinary fH. Here, we extend our evaluation to other proteins, namely, P, CR1 and MAC. We found that levels of urinary MAC, fH and P in patients with IgAN were significantly higher than those in healthy controls. Furthermore, urinary MAC and fH levels were significantly increased with increasing disease severity. Urinary MAC levels reflected disease state in patients with IgAN, as is the case in other nephropathies [24]. Urinary fH and P, which are involved in the regulation and stabilization of the alternative pathway C3 convertase, might also be associated with renal damage. Although only 4 patients had taken steroids when they were collected the urine samples, results of complement components presented not particular tendency.

In renal disease, renal function is closely related to tubulo-interstitial injury, part of which is due to MAC formation on tubular epithelial cells [25]. Indeed, urinary fH and P levels were strongly correlated with u-NAG and might reflect the occurrence of intra-tubular activation of C3. In additional work, we did find significant correlations between urinary fH and P (p < 0.001), fH and MAC (p < 0.001), and P and MAC (p < 0.001) (data not shown).

The human fH family consists of seven multi-domain and multifunctional serum proteins, including fH itself (MW 150 kDa), factor H-like protein 1 (FHL-1) (MW 42 kDa) and five factor H-related proteins (FHR-1, -2, -3, -4 and -5) [26]. This study demonstrated that fH, a 150 kDa protein, was detected in patients' urine samples, but lower MW members of this family were not detected in IgAN, with the exception of one patient with FHL-1. Because human mesangial cells and proximal tubular epithelial cells are capable of producing fH [27,28], urinary fH might be derived from glomeruli and/or tubules, not from the blood.

Previous studies showed that synthesis of membrane-bound CR1 on the podocytes is reduced in patients with advanced glomerular disease [29,30]. Urinary CR1 was released from podocytes, and did not originate from erythrocyte CR1 and soluble CR1 [31]. Likewise, as established here, urinary CR1 in patients with IgAN was significantly lower than in healthy controls.

There was a significant correlation between interstitial fibrosis and the percentage of global glomerular sclerosis as a fraction of all glomruli (p < 0.01). Thus, tubulointerstitial damage may be affected by glomerular injury. There were significant correlations between interstitial fibrosis and urinary protein, and between the percentage of global glomerular sclerosis and urinary protein. It was previously considered that the presence of urinary protein reflected glomerular damage and interstitial fibrosis. Therefore, interstitial fibrosis and the percentage of global glomerular sclerosis might be a marker of renal damage. Urinary fH and MAC were significantly correlated with interstitial fibrosis and the percentage of global glomerular sclerosis. Moreover, MAC showed a better correlation with interstitial fibrosis than did urinary protein, and fH correlated better with global sclerosis than urinary protein in patients with IgAN. Urinary fH showed a better correlation with serum creatinine, urinary NAG, urinary protein and global sclerosis than urinary MAC in patients with IgAN. It is proposed that in fact, fH might not regulate complement activation and subsequent formation of MAC. Therefore, MAC formation and renal damage might occur in IgAN. Further research is needed to clarify whether the relationships between urinary fH and complement activation might recapitulate in other types of glomerulonephritis.

## Conclusions

Complement activation occurs in the urinary space in IgAN and the measurement of levels of MAC and fH in the urine could be a useful indicator of renal injury in patients with IgAN.

## Competing interests

We (Kisara Onda, Isao Ohsawa, Hiroyuki Ohi, Mariko Tamano, Satoshi Mano, Michiro Wakabayashi, Akie Toki, Satoshi Horikoshi, Teizo Fujita and Yasuhiko Tomino) declare no conflict of interest in this study.

## Authors' contributions

KO collected samples, carried out the study, analyzed the data and wrote the manuscript. IO and HO principal investigator advised on the study and reviewed the manuscript. MT advised the experimental methods. SM and MW helped to collect samples. AT helped evaluation of fibrotic change and glomerular sclerosis. SH participated in the design of the study. TF gave us rabbit antisera to human fH, P, CR1 and purified P. YT primary principal investigator advised on the study. All authors have read and approved the final manuscript.

## Pre-publication history

The pre-publication history for this paper can be accessed here:

http://www.biomedcentral.com/1471-2369/12/64/prepub
